# Application of Trehalose and Salicylic Acid Mitigates Drought Stress in Sweet Basil and Improves Plant Growth

**DOI:** 10.3390/plants10061078

**Published:** 2021-05-27

**Authors:** Faisal Zulfiqar, Jianjun Chen, Patrick M. Finnegan, Adnan Younis, Muhammad Nafees, Walid Zorrig, Karim Ben Hamed

**Affiliations:** 1 Department of Horticultural Sciences, Faculty of Agriculture and Environment, The Islamia University of Bahawalpur, Bahawalpur 93100, Pakistan; Muhammad.nafees@iub.edu.pk; 2 Environmental Horticulture Department and Mid-Florida Research and Education Center, IFAS, University of Florida, 2725 Binion Road, Apopka, FL 32703, USA; jjchen@ufl.edu; 3 School of Biological Sciences, University of Western Australia, 35 Stirling Highway, Perth, WA 6009, Australia; patrick.finnegan@uwa.edu.au; 4 Institute of Horticultural Sciences, University of Agriculture Faisalabad, Faisalabad 38000, Pakistan; adnanyounis@uaf.edu.pk; 5 Laboratory of Extremophile Plants, Centre of Biotechnology of Borj-Cedria, P.O. Box 901, Hammam-Lif 2050, Tunisia; zorigwalid@gmail.com (W.Z.); karimbenhamed2016@gmail.com (K.B.H.)

**Keywords:** antioxidants, osmolytes, malondialdehyde, hydrogen peroxide, chlorophyll fluorescence, leaf gas exchange, phenolic compounds

## Abstract

Trehalose (Tre) and salicylic acid (SA) are increasingly used to mitigate drought stress in crop plants. In this study, a pot experiment was performed to study the influence of Tre and SA applied individually or in combination on the growth, photosynthesis, and antioxidant responses of sweet basil (*Ocimum basilicum* L.) exposed to drought stress. Basil plants were watered to 60% or 100% field capacity with or without treatment with 30 mM Tre and/or 1 mM SA. Drought negatively affected growth, physiological parameters, and antioxidant responses. Application of Tre and/or SA resulted in growth recovery, increased photosynthesis, and reduced oxidative stress. Application of Tre mitigated the detrimental effects of drought more than SA. Furthermore, co-application of Tre and SA largely eliminated the negative impact of drought by reducing oxidative stress through increased activities of antioxidant enzymes superoxide dismutase, peroxidase, and catalase, as well as the accumulation of the protective osmolytes proline and glycine betaine. Combined Tre and SA application improved water use efficiency and reduced the amount of malondialdehyde in drought-stressed plants. Our results suggested that combined application of Tre and SA may trigger defense mechanisms of sweet basil to better mitigate oxidative stress induced by drought stress, thereby improving plant growth.

## 1. Introduction

Under the threat of climate change, crop production is predicted to become more challenging due to increasing severity of various abiotic stresses [1,2]. Among them, drought stress is the leading ecological constraint to plant growth. It is the most critical concern, especially in the face of current scenarios of global warming [1], and it is predicted to become more frequent in numerous regions of the world [3].

Drought stress triggers an array of molecular, biochemical, physiological, anatomical, and morphological responses that have negative effects on plant growth and development [4,5,6,7]. In particular, a reduction in electron transport chain (ETC) activity during drought stress leads to accumulation of reactive oxygen species (ROS) that are toxic at elevated levels [8]. These oxidizing compounds react with and damage nucleic acids, proteins, photosynthetic pigments, and membrane lipids [9]. Plants have natural defense systems comprising non-enzymatic and enzymatic antioxidants that efficiently ameliorate the negative impacts of excessive ROS [9]. Additionally, plants acclimate to ROS-induced stress by producing various beneficial compatible solutes, such as proline and glycine betaine [10,11].

Osmoprotectants synthesized by plants play an important role in regulating defense responses to various abiotic stresses, including drought stress [12,13]. Recently, exogenous application of osmoprotectants has been widely adopted as an effective, economical, and eco-friendly strategy to manage various environmental extremes [10,14,15]. Trehalose (α-d-glucopyranosyl-[1,1]-α-d-glucopyranoside (Tre)) is a crucial non-reducing disaccharide, composed of two glucose units attached through their 1-carbons [16]. It acts as a cytoprotective agent under unfavorable environmental conditions [17,18]. Its chemical non-reactivity and high solubility allow it to accumulate to high concentrations without disrupting normal metabolism [19,20]. Thus, Tre acts as a both an osmoprotectant and a carbon storage and transport compound [17,18]. The beneficial role of Tre in ameliorating drought stress is related to its ability to enhance antioxidant systems, improve cellular redox balance, and promote photosynthesis [14,18,19]. Trehalose is also an osmoprotectant during dehydration and desiccation stress. It stabilizes biological structures by crystallizing into a glassy state that resists dehydration [18]. Furthermore, Tre is inexpensive and widely available, as well as being readily taken up by plants when exogenously applied [14,19,20,21]. However, it is not yet clear whether this strategy of producing Tre during drought stress is universal or effective in all plants.

Salicylic acid (SA) is an inexpensive phenolic compound that promotes plant growth due to its function as a plant hormone [22,23]. The endogenous biosynthesis of SA by plants changes under drought stress [24]. In view of this, several studies have reported that exogenous application of SA had a positive effect on plant performance under various abiotic stresses [25,26,27].

Sweet basil (*Ocimum basilicum* L.) is native to subtropical and tropical regions of Africa, Asia, and South America [28]. It is a popular and valuable horticultural plant that is used worldwide as a medicinal, ornamental, and culinary herb [29,30,31,32]. Sweet basil is marketed in both dry and fresh forms in the spice and food industries [18] and is used to produce essential oil on an industrial scale [33]. The oil is rich in phenolics, such as rosmarinic and caffeic acid [34], and it is used in perfume, cosmetics, and pharmaceuticals [18]. The oil contains aromatic components and volatile oils with antibacterial, antifungal, and nematicidal activities, and it is also an insect repellent due to an array of compounds [18].

Basil is an annual, herbaceous plant that grows best in warm and sunny conditions [27]. However, it is quite sensitive to drought stress [27,32]. The objectives of this study were to investigate whether exogenous application of Tre and SA, individually or in combination, could ameliorate drought stress by trigging oxidative stress resistance and improve growth of sweet basil.

## 2. Materials and Methods

### 2.1. Experimental Conditions

A pot experiment was conducted in outdoor (ambient) conditions using a commercially available sweet basil (*O. basilicum* L.) cv. ‘Lime Basil’. Uniform seeds were surface sterilized by treating with 4% (*w*/*v*) sodium hypochlorite for 15 min. Sterilized seeds were rinsed three times with deionized water. Ten seeds were placed in 1.25 L plastic pots containing a sandy loam soil composed of 60% sand, 29.5% silt, and 10.5% clay by volume with an electrical conductivity (EC) of 1.88 dS·m^−1^, pH 7.5, and a saturation percentage of 30%. After germination, seedlings at the cotyledon stage were thinned to five similarly sized seedlings per pot. One month after seed germination, plants were exposed to different watering levels by maintaining 100% (control) or 60% field capacity (FC). Optimum FC was established on soil saturation percentage. After 20 days of maintaining these two watering levels, 30 mM Tre and 1 mM SA alone or in combination (Tre + SA) were applied twice at 5 day intervals. Tween-20 (0.01 mL/L) was added to the Tre and SA solutions as a surfactant and spreading facilitator. Soil water status was maintained for a further two weeks after the second chemical treatment when plants were harvested. The average climatic conditions over the entire experiment were 22/17 °C day/night temperatures, 16 h photoperiod, and 60–65% relative humidity. The experiment was set in a completely randomized design with six replications.

### 2.2. Leaf Characteristics

Leaf tissue was sampled for chlorophyll (Chl) analysis 4 days before the end of the experiment. Chlorophyll was extracted and its concentration calculated using the method and equations described by Arnon [35]. Freshly harvested leaf material was immediately cut into small pieces (approximately 0.5 cm^2^) with scissors. Leaf material (approximately 0.5 g per plant) was extracted with 10 mL 80% (*v*/*v*) acetone at −4 °C for 12 h. The leaf extract was clarified by centrifugation (ALC centrifuge PK130R) at 14,000× *g* for 5 min at room temperature. Absorbance of the extract was recorded spectrophotometrically at 645 and 663 nm (SmartSpecTM Plus, BioRad) for chlorophyll *a* (Chl-*a*) and chlorophyll *b* (Chl-*b*), respectively. 

The leaf gas exchange parameters net photosynthesis (*A*; μmol CO_2_·m^−2^·s^−1^), transpiration rate (*E*; mmol H_2_O·m^−2^·s^−1^), intercellular CO_2_ concentration (*Ci*; cm^3^·m^−3^), and stomatal conductance (*gs*; mmol H_2_O·m^−2^·s^−1^) were recorded using a portable carbon dioxide (CO_2_) infrared gas analyzer (GFS-3000FL, Heinz Walz GmbH, Effeltrich, Germany). During measurements, the air temperature was 32.3 to 35.1 °C, with a relative humidity of 61%, ambient pressure of 98.01 kPa, and photosynthetic photonflux density of 1200 μmol·m^−2^·s^−1^. Measurements were taken on young fully expanded leaves on a sunny day between 10:00 a.m. and 12:30 p.m.

The chlorophyll fluorescence parameters photosystem efficiency (Fv/Fm), photochemical quenching (qP), coefficient of non-photochemical quenching (qN), and non-photochemical quenching (NPQ) were measured using a chlorophyll fluorometer (FMS2, Hansa Tech Ltd., King’s Lynn, UK).

### 2.3. Leaf Total Phenolic Concentration

Total phenolics were measured following Julkenen-Titto [36]. Fresh leaves (0.5 g) were minced and mixed with 1 mL 80% (*v*/*v*) acetone and centrifuged (ALC centrifuge PK130R) at 12,000× *g* for 10 min at room temperature. Samples were then incubated in an ice bath for 15 min. A 100 µL aliquot of the supernatant was mixed with 2 mL of distilled water and 1 mL of Folin–Ciocâlteu’s phenol reagent. After vigorously shaking the mixture, 5 mL of 20% (*w*/*v*) Na_2_CO_3_ was added, and the final volume was brought to 10 mL with distilled water. After 20 min at ambient temperature, the absorbance of the solution was recorded spectrophotometrically at 750 nm following Julkenen-Titto [36].

### 2.4. Electrolyte Leakage

A fresh leaf of about 200 mg was washed twice with distilled water, cut into small uniform pieces with scissors, and placed in 10 mL of distilled water. Samples were incubated in a water bath for 2 h at 32 °C. The initial electrical conductivity reading (EC1) of the solution was measured using an electrical conductivity meter (LF 538, WTW GmbH, Weilheim, Germany). Each sample was then autoclaved at 121 °C for 20 min and cooled to 25 °C, before the final electrical conductivity (EC2) was measured. Electrolyte leakage was determined using the formula developed by Dionisio-Sese and Tobita [37].

Electrolyte leakage (%) = (EC1/EC2) × 100.

### 2.5. Osmolyte Quantification

The concentration of leaf free proline (Pro) was determined as described by Bates et al. [38], where 0.5 g of fresh leaf material was homogenized in 10 mL 3% (*w*/*v*) sulfosalicylic acid, and the homogenate was centrifuged at 4000× *g* for 10 min. A 2 mL aliquot of supernatant was supplemented with 2 mL of acidic ninhydrin solution (1.26 g of ninhydrin + 20 mL of 6 M *ortho*-phosphoric acid + 30 mL of glacial acetic acid) and 2 mL of glacial acetic acid. The mixture was incubated at 100 °C for 60 min. After incubation, 4 mL of toluene was added and mixed by shaking for extraction. The chromophore-containing toluene was aspirated and cooled to room temperature. The absorbance was recorded spectrophotometrically at 520 nm (SmartSpecTM Plus, BioRad). Proline concentration was calculated by comparing with standard curve constructed using known concentrations of Pro.

Glycine betaine (GB) concentration in leaves was measured following the methodology of Grieve and Grattan [39]. For GB extraction, 0.5 g of leaf tissue was homogenized in 20 mL of distilled water at room temperature and then incubated at 4 °C overnight. The extracts were clarified by centrifugation at 10,000× *g* for 5 min at ambient temperature. A 1 mL aliquot of supernatant was diluted with 1 mL of 2 N H_2_SO_4_ and 0.2 mL of 1 M KI_3_. Samples were cooled for 1.5 h in an ice bath before adding 2.8 mL of distilled water and 6 mL of 1,2-dichloroethane in periodic crystals. After phase separation, the upper aqueous phase was discarded, and the absorbance of the lower organic phase was read at 365 nm (SmartSpecTM Plus spectrophotometer, BioRad, Hercules, CA, USA). The concentrations of GB were determined against a standard curve following Grieve and Grattan [39].

### 2.6. Determination of Hydrogen Peroxide

The methodology developed by Velikova et al. [40] was used to determine hydrogen peroxide (H_2_O_2_). Fresh leaf material (200 mg) was crushed under liquid nitrogen followed by homogenizing in 1.5 mL 0.1% (*w*/*v*) trichloroacetic acid (TCA). The homogenate was centrifuged at 12,000× *g* for 15 min at 4 °C. A 0.5 mL aliquot of supernatant was supplemented with 0.5 mL of 10 mM potassium phosphate (pH 7) and 1 mL of 1 M KI, followed by vortex mixing. The H_2_O_2_ concentration was determined by reading the absorbance at 390 nm (SmartSpecTM Plus spectrophotometer, BioRad) and using a standard curve.

### 2.7. Determination of Malondialdehyde

Malondialdehyde (MDA) concentration was measured to estimate lipid peroxidation using the protocol developed by Heath and Packer [41]. Briefly, 0.25 g of minced leaf sample was mixed in 5 mL of 0.5% (*w*/*v*) thiobarbituric acid. The mixture was incubated at 95 °C in a water bath for 50 min and then cooled in an ice bath. The MDA concentration in the supernatant was determined by measuring absorbance at 600 nm and 532 nm (SmartSpecTM Plus spectrophotometer, BioRad).

### 2.8. Antioxidant Enzyme Extraction and Assay

Fresh leaf material (0.5 g) was homogenized in a chilled mortar and pestle containing 5 mL of ice-cold 50 mM sodium phosphate buffer, pH 7.8, containing 2% (*w*/*v*) polyvinylpyrrolidone and 1.0 mM EDTA. The homogenate was centrifuged (Z 306; HERMLE Labortechnik, Wehingen, Germany) at 10,000× *g* for 20 min at 4 °C. The supernatant was stored at −20 °C, and catalase (CAT), peroxidase (POD), and superoxide dismutase (SOD) activities were determined as described below.

Catalase and Peroxidase activity were estimated using the method of Chance and Maehly [42]. Superoxide dismutase activity was assayed using the method of Van Rossum et al. [43]. 

### 2.9. Plant Vegetative Characteristics

At the termination of the experiment, six plants from each treatment were uprooted carefully for growth analysis. After washing with tap water, plants were divided into shoot and root portions. Shoot and root length and fresh mass were measured. Plant parts were placed separately in paper bags and dried at 70 °C for 72 h before measuring dry mass.

### 2.10. Statistical Analyses

All data were subjected to analysis of variance (ANOVA) using XLSTAT program, version 2014 (Addinsoft, New York, NY, USA). Mean separation for significant differences was done using Duncan’s multiple-range tests at the *p* < 0.01 level.

## 3. Results

### 3.1. Plant Growth Responses

**Figure 1:**
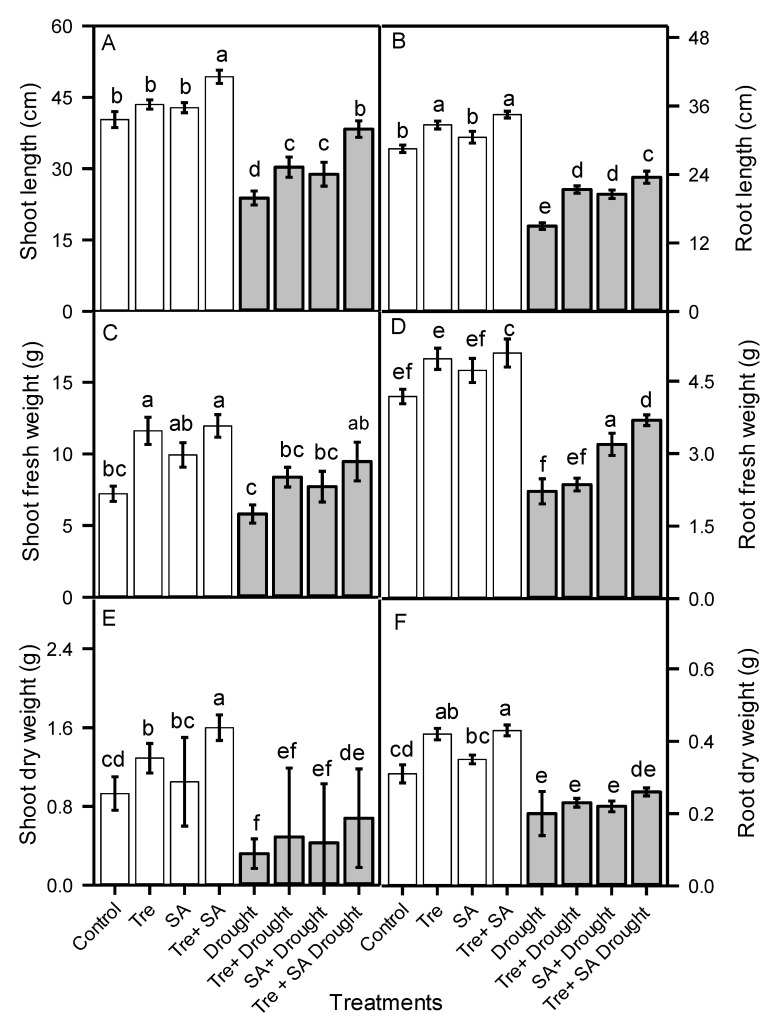
Shoot and root lengths (**A**,**B**), fresh and dry weight (**C**–**F**), of sweet basil (*Ocimum basilicum*) after foliar application of trehalose and salicylic acid individually or in combination during drought stress. One month old seedlings were watered to 100% (control, open bars) or 60% (drought, filled bars) field capacity (FC) for 20 days. Trehalose (Tre) and/or salicylic acid (SA) were then applied twice with a 5 day interval between applications. The watering conditions were maintained for an additional 2 weeks until plants were harvested. Data are the means ± SE (*n* = 6). Different letters above the bars represent significant differences according to Duncan’s multiple-range test at the *p* < 0.01 level.

Shoot length in well-watered sweet basil treated with Tre or SA alone was about 5% longer than in untreated control plants (Figure 1). Combining Tre + SA had a synergistic effect, producing plants with shoots that were 16% longer than in the untreated control. Drought stress (60% FC) reduced both shoot and root length by about 50% (Figure 1). The shoots of drought-stressed plants sprayed with Tre or SA were about 20% longer, compared to unsprayed drought-subjected plants. Moreover, roots were about 30% longer in drought-subjected seedlings treated with Tre or SA, compared to untreated drought-stressed plants. The combined application of Tre + SA to drought-stressed plants resulted in shoots and roots that were 39% and 36% longer, respectively, than in unsprayed drought-stressed plants.

**Figure 2:**
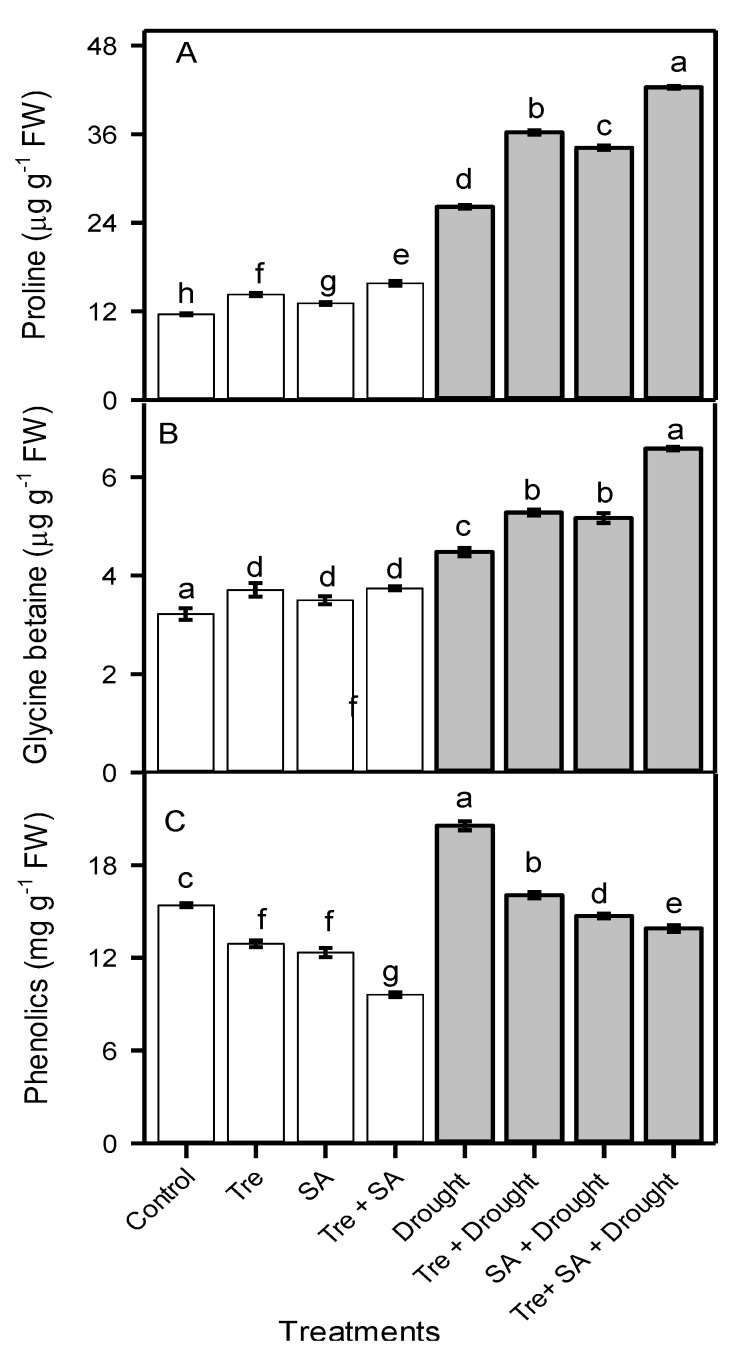
Leaf concentrations of proline (**A**), glycine betaine (**B**), and total phenolics (**C**) in sweet basil (*Ocimum basilicum*) after foliar application of 30 mM trehalose (Tre) and/or 1 mM salicylic acid (SA) during drought stress. One month old seedlings were watered to 100% (control) or 60% (drought) field capacity for 20 days. Tre and/or SA were then applied twice with a 5 day interval. The watering conditions were maintained for an additional 2 weeks until plants were harvested. Data are the means ± SE (*n* = 6). Different letters above the bars indicate significant differences according to Duncan’s multiple-range test at the *p* < 0.01 level.

Shoot fresh mass and dry mass were reduced 34% and 77%, respectively, in drought-stressed seedlings compared to control plants (Figure 1). Drought-stressed seedlings treated with Tre or SA had enhanced shoot fresh and dry mass compared to drought-stressed plants that were left untreated. The combined application of Tre + SA to drought-stressed seedlings increased shoot fresh mass by 37% compared to drought-stressed plants that were left untreated (Figure 2), and it increased shoot dry mass by 71% (Figure 1).

Drought stress decreased root fresh and dry mass by 50% and 36%, respectively, compared to well-watered control plants. Application of Tre and SA alone had little effect on the fresh or dry mass of roots. However, application of Tre + SA to drought-stressed plants did enhance root fresh mass (Figure 1), but to a lesser extent than in shoot.

### 3.2. Leaf Chlorophyll Concentration and Leaf Gas Exchange

Leaf Chl-*a* concentration in plants grown at 100% FC increased by 6%, 4%, and 14% after application of Tre, SA, and Tre + SA, respectively (Table 1). Drought stress caused a decline in Chl-*a* by 43% compared to the control. However, supplementing drought-stressed plants with Tre or SA increased Chl-*a* concentration by 43% and 28%, respectively, relative to untreated drought-stressed plants. The combined application of Tre + SA to drought-stressed plants caused a 61% increase in Chl-*a* concentration compared to untreated drought-stressed plants (Table 1).

Leaf Chl-*b* concentration in well-watered (100% FC) plants increased by 13%, 7%, and 25% after exogenous application of Tre, SA, and Tre + SA, respectively (Table 1). Drought stress caused a marked decline of 39% in Chl-*b* concentration relative to well-watered plants (Table 1). Drought-stressed plants treated with Tre or SA had Chl-*b* concentrations that were 29% and 15% higher, respectively, than plants subjected to drought alone. The combined application of Tre + SA to drought-stressed plants caused a 44% increase in the Chl-*b* concentration above that of the untreated drought-stressed plants.

Total Chl concentration in Tre, SA, and Tre + SA treated plants watered to 100% FC was 8%, 5%, and 16% higher, respectively, than in the untreated control plants. Drought stress caused a 42% decline in total Chl concentration compared to the well-watered control (Table 1). However, total Chl concentrations in drought-stressed plants treated with Tre or SA were 41% and 26% higher, respectively, than in the untreated drought-stressed plants. The application of Tre + SA to drought-stressed plants increased the total Chl concentration by 57% compared to untreated drought-stressed plants (Table 1).

The application of Tre or SA, alone or in combination, to well-watered plants increased the leaf gas exchange characteristics *A*, *gs*, *E*, and *Ci* compared to the untreated control (Table 2). In contrast, drought stress decreased *A*, *gs*, *E*, and *Ci* by 49%, 80%, 81%, and 37%, respectively, compared to the well-watered control. Treatment of drought-stressed plants with Tre enhanced *A* by 47%, *gs* by 139%, *E* by 88%, and *Ci* by 26% relative to untreated drought-stressed plants. The application of SA resulted in a similar level of amelioration of these parameters. Combining Tre + SA led to increases in *A*, *gs*, *E*, and *Ci* of 66%, 420%, 160%, and 38%, respectively, compared to untreated drought-stressed plants (Table 2).

### 3.3. Chlorophyll Fluorescence

Treatment of well-watered plants with Tre or SA, alone or in combination, had little impact on PSII efficiency (Fv/Fm), PSII quantum yield (ΦPSII), or photochemical efficiency (qp) (Table 3). However, both Tre and SA treatments caused a decrease in nonphotochemical quenching (NPQ) (Table 3). When both chemicals were applied together, the decrease in NPQ was 44% of the value in untreated plants. Drought stress reduced Fv/Fm by 31%, ΦPSII by 25%, and qp by 31%, compared to control plants, but increased NPQ by 51%. Application of Tre to drought-stressed plants increased Fv/Fm by 22%, ΦPSII by 20%, and qp by 18%, and decreased NPQ by 23% relative to untreated drought-stressed plants. Treating drought-stressed plants with SA enhanced Fv/Fm, ΦPSII, and qp by about 12% for each and decreased NPQ by 14% relative to untreated drought-stressed plants (Table 3). The combination of Tre + SA in drought-stressed plants caused an increase in Fv/Fm and ΦPSII of about 65%, an increase in qp of 35%, and a decrease in NPQ of 28%, compared to untreated drought-stressed plants (Table 3).

### 3.4. Leaf Free Proline and Glycine Betaine Concentrations

Drought stress increased the leaf free Pro and GB concentrations by 126% and 39%, compared to the well-watered control plants (Figure 2). Exogenous application of Tre further increased the leaf free Pro concentration by 38% and GB concentration by 18% compared to untreated drought-stressed plants. Foliar application of SA increased the Pro concentration by 23% and GB concentration by 13% relative to unsprayed drought-stressed plants. The combination of Tre + SA increased the Pro concentration by 62% and GB concentration by 46% compared to untreated plants under drought stress. Drought stress increased total phenolic concentration (TPC) by 33% relative to control plants. However, exogenous application of Tre, SA, and Tre + SA to drought-stressed seedlings caused a reduction in TPC of 22%, 28%, and 27%, respectively, compared to untreated drought-stressed plants (Figure 2).

### 3.5. Electrolyte Leakage, and Hydrogen Peroxide and Malondialdehyde Concentrations

**Figure 3:**
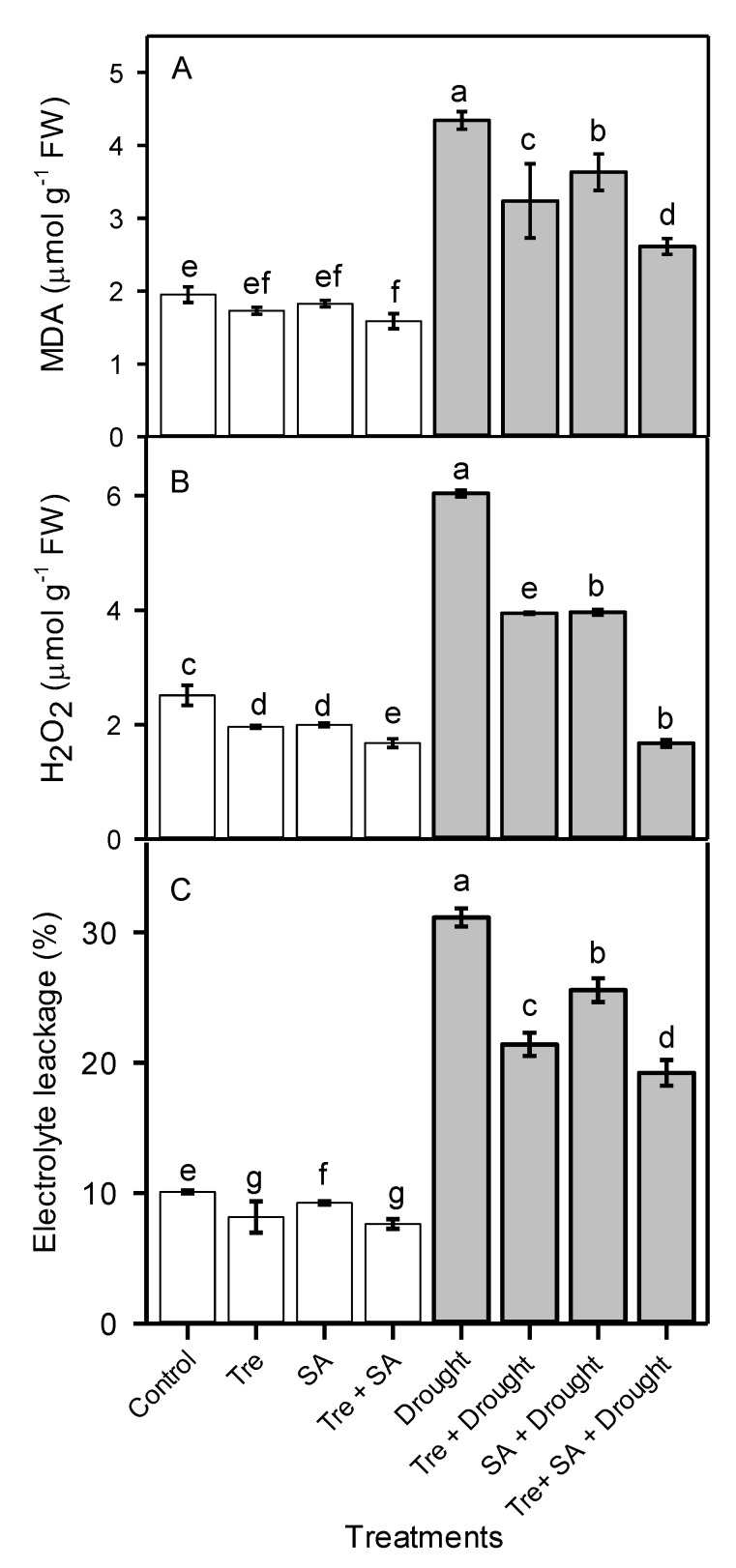
Malondialdehyde concentration (MDA) (**A**), hydrogen peroxide concentration (H_2_O_2_) (**B**), and electrolyte leakage (EL) (**C**) of sweet basil (*Ocimum basilicum*) after foliar application of 30 mM trehalose (Tre) and/or 1 mM salicylic acid (SA) during drought stress. One month old seedlings were watered to 100% (control) or 60% (drought) field capacity for 20 days. Tre and/or SA were then applied twice with a 5 day interval. The watering conditions were maintained for an additional 2 weeks until plants were harvested. Data are the means ± SE (*n* = 6). Different letters above the bars indicate significant differences among means according to Duncan’s multiple-range test at the *p* < 0.01 level.

Drought stress increased malondialdehyde (MDA) and H_2_O_2_ concentrations, as well as electrolyte leakage (EL), by 2.3-fold, 2.7-fold, and 3.2-fold, respectively, compared to the well-watered control (Figure 3). However, in drought-stressed plants, exogenous application of Tre decreased MDA and H_2_O_2_ concentrations by 25% and 34%, respectively, and EL by 32%, relative to the untreated drought-stressed plants. Similarly, application of SA reduced MDA and H_2_O_2_ concentrations by 16% and 34%, respectively, and EL concentration by 18%, relative to untreated drought-stressed plants. The combined application of Tre + SA was more effective in alleviating the increases in MDA and H_2_O_2_ concentrations and EL induced by drought stress than the separate applications of the compounds. The Tre + SA treatment reduced MDA and H_2_O_2_ concentrations and EL by 40%, 72%, and 38%, respectively, relative to untreated drought-stressed plants (Figure 3).

### 3.6. Activity of Antioxidant Enzymes

**Figure 4:**
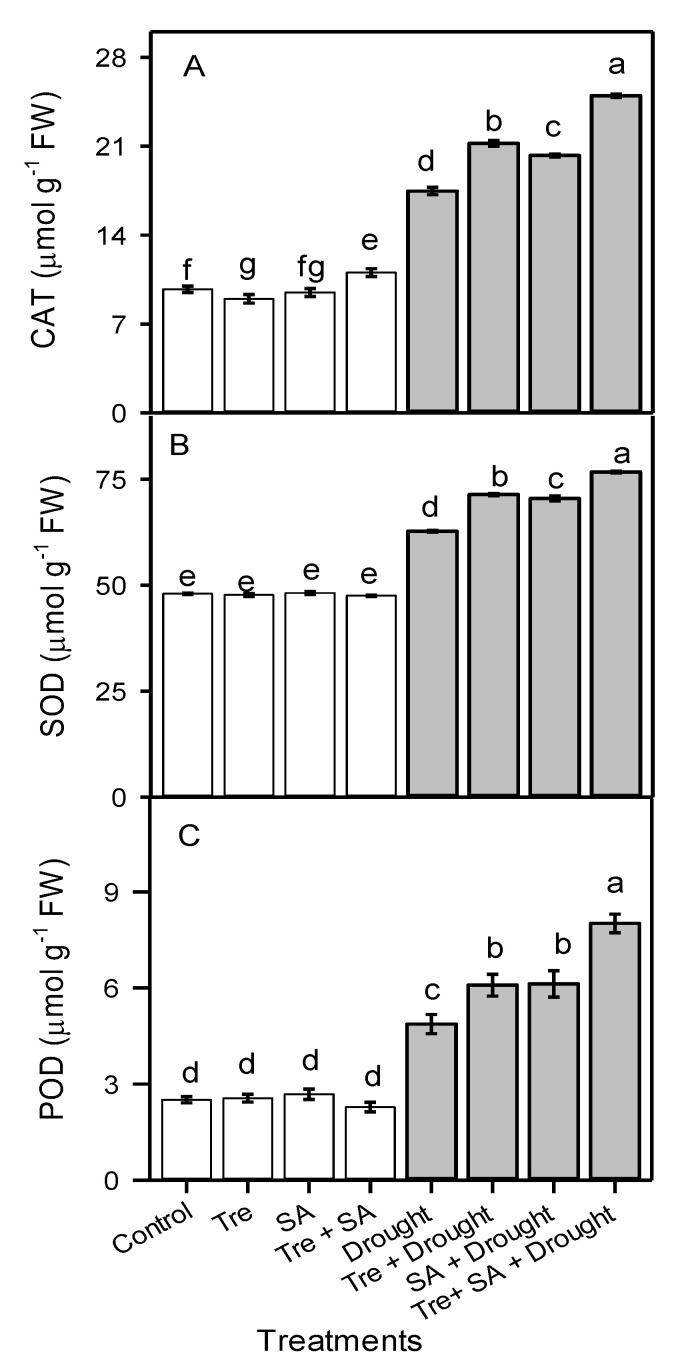
Catalase (CAT) (**A**), superoxide dismutase (SOD) (**B**), and peroxidase (POD) (**C**) activities in sweet basil (*Ocimum basilicum*) after foliar application of trehalose (Tre) and/or salicylic acid (SA) during drought stress. One month old seedlings were watered to 100% (control) or 60% (drought) field capacity for 20 days. Tre and/or SA were then applied twice with a 5 day interval. The watering conditions were maintained for an additional 2 weeks until plants were harvested. Data are the means ± SE (*n* = 6). Different letters above the bars indicate significant differences among means according to Duncan’s multiple-range test at the *p* < 0.01 level.

Drought stress induced CAT, SOD, and POD activities by 79%, 31%, and 94%, respectively, compared to well-watered control seedlings (Figure 4). Application of Tre further increased CAT activity by 21%, SOD activity by 14%, and POD activity by 25% in drought-stressed seedlings over untreated drought-stressed seedlings. Foliar application of SA increased CAT, SOD, and POD activities by 16%, 12%, and 26%, respectively, compared to untreated drought-stressed plants. The combined application of Tre + SA increased CAT activity by 43%, SOD activity by 22%, and POD activity by 60% relative to untreated drought-stressed seedlings (Figure 4).

## 4. Discussion

Drought stress reduced all the vegetative growth parameters of sweet basil seedlings that were tested, as earlier reported by Damalas [27]. In the present study, the decreased growth of sweet basil under drought was probably due to the disturbance of various vital metabolic functions associated with chlorophyll biosynthesis, photosynthesis, and oxidative metabolism.

Application of Tre and/or SA mitigated the drought effects in basil through the partial recovery of chlorophyll levels and photosynthetic activity. Similar results have been reported in several plant species, such as sunflower [44], sesame [45], sweet basil [27], squash [46], *Eucalyptus globulus* L. [47], and maize (*Zea mays* L.) [48]. SA and Tre probably acted through the maintenance of the structural integrity of the thylakoid membrane and protected PSII from over-excitation and oxidative stress, as clearly shown by the increased Fv/Fm ratio and the reduced NPQ, especially when Tre and SA were used in combination. Similar effects were reported in *E. globulus* [47]. Taken together, our results support a role for SA and Tre in the protection of photosynthetic apparatus [44] through increased chlorophyll synthesis and photosynthetic activity, which support increased plant growth during drought stress.

Electrolyte leakage, H_2_O_2_, and MDA are considered to be oxidative stress markers in plants under extreme environmental conditions. Sweet basil plants subjected to drought stress showed increased EL, potentially due to increased H_2_O_2_ and MDA concentrations, compared to well-watered control plants. This result was consistent with previous studies in safflower (*Carthamus tinctorius* L.) [48] and *E. globulus* [47]. Spraying the foliage of drought-stressed sweet basil plants with Tre and/or SA reduced H_2_O_2_ and MDA concentrations, as well as EL, indicating that Tre and SA alleviate the oxidative stress associated with drought stress. The results agree with previous studies in *Brassica rapa* L. [49] and wheat (*Triticum aestivum* L.) [50]. SA and Tre are likely to act in membrane protection, as well as in scavenging toxic reactive oxygen species produced during oxidative stress. Damalas [27] reported that a distinct characteristic in SA-treated sweet basil was elevated relative water content (RWC) in leaves. The increased RWC may indicate that SA enhanced membrane protection during drought stress.

The decrease in the magnitude or concentration of the oxidative stress indicators MDA, H_2_O_2_, and EL after SA and/or Tre application suggested an increase in antioxidant activity in basil to protect the plant against toxicity due to the production of drought-induced ROS toxicity. Generally, antioxidant systems in plants involve antioxidant enzymes, such as SOD, CAT, nonspecific POD, and ascorbate peroxidase, as well as non-enzymatic molecules such as ascorbate, glutathione, phenols, and osmolytes. In the present study, antioxidant enzyme activities were induced in response to drought stress and were further induced in stressed plants by the application of Tre and SA. A similar effect of SA on antioxidant enzyme activity under drought stress has been reported in safflower [48], *B. rapa* [49], and wheat [50]. Contrary to the effect on antioxidant enzymes, treatment of drought-stressed plants with SA and Tre caused a decrease in total phenol concentration. Phenols have been well documented to protect plants under stress by scavenging hydroxyl radicals [51]. Drought stress has been reported to either increase or decrease phenol concentrations depending on plant species [51,52]. The lower concentrations of phenols after the application of Tre and/or SA in drought-stressed basil suggested that they have only a weak involvement in tolerance to drought stress in this plant, unlike antioxidant enzymes. The mechanism through which Tre and SA caused a decrease in the concentrations of phenols needs further investigation.

Proline and GB are other multifunction molecules that protect plant membranes against dehydration and oxidation [53,54]. Proline has many functions under harsh environmental conditions. The most pronounced of these functions are to protect the photosynthetic machinery, enzymes, and membranes, to be used as an energy storage compound, and to provide resistance against cellular dehydration, as well as to act as a molecular chaperone. Together, these functions contribute to stress tolerance without damaging cellular structure. Additionally, Pro helps reduce oxidative stress in plants by enhancing the activities of ROS-scavenging antioxidants that decrease toxic ROS levels [55]. Similarly, GB functions in ROS detoxification, osmoregulation, maintenance of membrane integrity, protection of plant photosynthetic apparatus, and modulating stress-related gene activation [56]. In the present study, Pro and GB levels increased in sweet basil seedlings subjected to drought stress, and these levels increased further after treatment with Tre and SA individually or in combination. Treatment with Tre increased Pro accumulation in various plant species under drought stress [43]. Ibrahim and Abdellatif [55] reported elevated Pro accumulation in wheat subjected to drought after treatment with Tre. The application of SA to sweet basil seedlings also enhanced Pro and GB levels under drought stress. Previous reports are in agreement with the current findings [27,48,49]. According to Nazar et al. [56], enhanced Pro production via increased γ-glutamyl kinase and reduced proline oxidase activity after SA application enhanced tolerance to drought stress.

## 5. Conclusions

The current study documented morpho-physiological and biochemical responses of sweet basil seedlings to drought stress and demonstrated that foliar application of Tre and/or SA mitigated the effects of drought stress. Drought stress caused increased production of ROS, leading to electrolyte leakage and lipid peroxidation. Applying Tre and/or SA to drought-stressed seedlings improved physiological characteristics and reduced ROS accumulation by enhancing the activities of antioxidant enzymes, which alleviated drought stress and enhanced plant growth. These findings demonstrated the comparative and combined potential of Tre, which is an osmolytic soluble sugar, and SA, which is a plant growth regulator, to reduce drought stress. These results will be helpful for developing strategies to mitigate drought-stress-induced oxidative stress and ultimately yield reductions in scenarios of climate change and a high demand for food production, especially in arid and semiarid regions.

## Figures and Tables

**Table 1:** Effect of foliar application of 30 mM trehalose (Tre) and/or 1 mM salicylic acid (SA) on chlorophyll *a* (Chl-*a*) and *b* (Chl-*b*), as well as total chlorophyll (Chl), concentrations in sweet basil seedlings grown under well-watered (control) and drought stress conditions. Values are the means ± SE (*n* = 6). Different letters within a column indicate significant differences among means according to Duncan’s multiple-range test at the *p* < 0.05 level.

Treatments	Chl-*a* (mg·g^−1^ FW)	Chl-*b* (mg·g^−1^ FW)	Total Chl (mg g^−1^ FW)
Control (CK)	1.75 ± 0.006 d	0.56 ± 0.003 d	2.31 ± 0.004 d
CK + Tre	1.87 ± 0.003 b	0.63 ± 0.006 b	2.50 ± 0.003 b
CK + SA	1.82 ± 0.003 c	0.60 ± 0.006 c	2.43 ± 0.007 c
CK + Tre + SA	1.99 ± 0.005 a	0.71 ± 0.009 a	2.70 ± 0.007 a
Drought stress	0.98 ± 0.007 h	0.34 ± 0.005 h	1.33 ± 0.007 h
Drought stress + Tre	1.43 ± 0.004 f	0.45 ± 0.003 f	1.87 ± 0.002 f
Drought stress + SA	1.28 ± 0.005 g	0.40 ± 0.006 g	1.67 ± 0.004 g
Drought stress + Tre + SA	1.60 ± 0.007 e	0.50 ± 0.009 e	2.10 ± 0.012 e

**Table 2:** Effect of foliar application of 30 mM trehalose (Tre) and/or 1 mM salicylic acid (SA) on leaf gas exchange traits in sweet basil seedlings grown under well-watered (control, CK) and drought stress conditions. Values are the means ± SE (*n* = 6). Different letters within a column indicate significant differences among means according to Duncan’s multiple-range test at the *p* < 0.05 level.

Treatments	CO_2_ Assimilation Rate (*A*) (µmol CO_2_·m^−2^·s^−1^)	Stomatal Conductance (*gs*) (mmol H_2_O·m^−2^·s^−1^)	Transpiration (*E*) (mmol H_2_O·m^−2^·s^−1^)	Intercellular CO_2_ conc. (*Ci*) (cm^3^·m^−3^)
Control (CK)	6.49 ± 0.13 d	53.00 ± 1.44 d	3.21 ± 0.017 c	222.67 ± 1.38 c
CK + Tre	7.71 ± 0.08 b	74.67 ± 1.38 b	3.89 ± 0.035 a	241.00 ± 2.35 b
CK + SA	7.26 ± 0.05 c	68.17 ± 3.79 c	3.72 ± 0.024 b	235.50 ± 2.09 b
CK + Tre + SA	8.74 ± 0.07 a	88.67 ± 2.79 a	3.98 ± 0.016 a	273.50 ± 2.96 a
Drought stress	3.29 ± 0.08 h	10.67 ± 0.61 g	0.61 ± 0.061 f	141.17 ± 2.15 g
Drought stress + Tre	4.85 ± 0.03 f	25.50 ± 0.81 f	1.13 ± 0.017 e	178.50 ± 3.42 e
Drought stress + SA	4.14 ± 0.02 g	23.83 ± 0.91 f	1.04 ± 0.039 e	169.17 ± 0.95 f
Drought stress + Tre + SA	5.49 ± 0.06 e	44.83 ± 1.19 e	1.57 ± 0.060 d	194.50 ± 2.36 d

**Table 3:** Effect of foliar application of 30 mM trehalose (Tre) and/or 1 mM salicylic acid (SA) on chlorophyll fluorescence parameters in sweet basil seedlings grown under well-watered (control, CK) and drought stress conditions. Values are the means ± SE (*n* = 6). Different letters within a column indicate significant differences among means according to Duncan’s multiple-range test at the *p* < 0.05 level.

Treatments	PSII Efficiency (Fv/Fm)	PSII Quantum Yield (FPSII)	Photochemical Quenching (qp)	Non-Photochemical Quenching (NPQ)
Control (CK)	0.80 ± 0.008 b	0.61 ± 0.02 b	0.87 ± 0.01 a	0.63 ± 0.01 d
CK + Tre	0.86 ± 0.003 a	0.68 ± 0.01 b	0.91 ± 0.009 a	0.44 ± 0.01 e
CK + SA	0.83 ± 0.007 a	0.63 ± 0.007 b	0.90 ± 0.013 a	0.39 ± 0.01 e
CK + Tre + SA	0.91 ± 0.011 a	0.73 ± 0.02 a	0.94 ± 0.016 a	0.35 ± 0.009 f
Drought stress	0.55 ± 0.004 d	0.46 ± 0.007 d	0.60 ± 0.006 d	0.95 ± 0.004 a
Drought stress + Tre	0.67 ± 0.020 c	0.55 ± 0.007 c	0.71 ± 0.010 c	0.73 ± 0.012 c
Drought stress + SA	0.62 ± 0.007 c	0.51 ± 0.008 c	0.67 ± 0.010 c	0.83 ± 0.025 b
Drought stress + Tre + SA	0.90 ± 0.012 a	0.76 ± 0.013 a	0.81 ± 0.005 b	0.68 ± 0.009 d

## Data Availability

The data presented in this study are available in the article.
